# Glycemic Alterations in Hospitalized COVID-19 Patients: Hyperglycemia and Newly Detected Diabetes

**DOI:** 10.3390/epidemiologia7020054

**Published:** 2026-04-13

**Authors:** Alecsandra Andreea Budihoi, Bogdana Nasui, Alexandra-Ioana Roșioară, Nina Ciuciuc, Stefan Vesa, Tudor Calinici, Monica Popa

**Affiliations:** 1 Department of Infectious Diseases and Epidemiology, Iuliu Hațieganu University of Medicine and Pharmacy, 400349 Cluj-Napoca, Romania; budihoi.alecsandra.andreea@elearn.umfcluj.ro; 2 Department of Community Medicine, Iuliu Hațieganu University of Medicine and Pharmacy, 400349 Cluj-Napoca, Romania; alexandra.rosioara@umfcluj.ro (A.-I.R.); nina.ciuciuc@umfcluj.ro (N.C.); monica.popa@umfcluj.ro (M.P.); 3 Research Center in Preventive Medicine, Health Promotion and Sustainable Development, Iuliu Hațieganu University of Medicine and Pharmacy, 400349 Cluj-Napoca, Romania; 4 Department of Pharmacology, Toxicology and Clinical Pharmacology, Iuliu Hațieganu University of Medicine and Pharmacy, 400349 Cluj-Napoca, Romania; stefan.vesa@umfcluj.ro; 5 Department of Medical Informatics and Biostatistics, Iuliu Hațieganu University of Medicine and Pharmacy, 400349 Cluj-Napoca, Romania; tcalinici@umfcluj.ro

**Keywords:** COVID-19, altered blood sugar, hyperglycemia, newly diagnosed diabetes, Cluj-Napoca, Romania

## Abstract

Background and Objective: The aim of this study is to describe the frequency of newly detected dysglycemia, including hyperglycemia and newly diagnosed diabetes mellitus, among hospitalized COVID-19 patients without previously known diabetes and to identify associated clinical and therapeutic factors, in an exploratory, descriptive manner. Materials and Methods: We conducted a retrospective study on 562 COVID-19 patients. Demographic and clinical data were collected at admission and during hospitalization. Newly diagnosed diabetes mellitus was defined based on plasma glucose values meeting international diagnostic criteria during hospitalization in patients without prior diabetes, while newly altered blood sugar referred to transient hyperglycemia or impaired fasting glucose not fulfilling diabetes criteria. Comparisons between groups were performed using appropriate statistical tests, with a *p*-value < 0.05 considered statistically significant. Results: Out of the total number of 562 COVID-19 patients, 14 (2.49%) were classified as having newly diagnosed diabetes, and 27 (4.8%) as having newly altered blood sugar levels. The median age of the participants was 67.5 years (interquartile range: 59.75; 71.75). Newly diagnosed diabetes was more frequently observed among patients presenting with gastrointestinal symptoms, elevated inflammatory markers, and those receiving specific in-hospital treatments. Newly altered blood sugar levels were more commonly associated with dyslipidemia, respiratory symptoms at admission, oxygen therapy, and selected COVID-19 treatments. COVID-19 vaccination status was descriptively stratified by admission period. Conclusions: New interdisciplinary approaches may support the identification and monitoring of glycemic alterations in hospitalized COVID-19 patients, with potential implications for clinical management and public health strategies.

## 1. Introduction

Diabetes is one of the most significant public health challenges, affecting millions of people worldwide. By 2040, the number of cases is projected to exceed 642 million. In Europe, the International Diabetes Federation (IDF) projects that the number of adults with diabetes will rise to 69 million by 2045 [1]. According to the World Health Organization’s (WHO) 2016 Diabetes Country Profile for Romania, there has been a slight increase in cases, particularly among men [2]. The prevalence of diabetes was reported at 8.5% in males and 8.4% in females. Key risk factors for diabetes include overweight (60.8%), obesity (23.4%), and physical inactivity (26.5%) [2].

In this context, disturbance of glucose metabolism, including newly diagnosed diabetes or dysglycemia, have been reported following hospitalization for other viral infections or acute illnesses. Numerous studies have reported cases of newly diagnosed diabetes, classified as either type 1 or type 2 diabetes, or COVID-19-associated hyperglycemia. A study from London, U.K., examined 30 children aged 23 months to 16.8 years with new-onset type 1 diabetes. Among them, 15% had a positive COVID-19 test. The study suggested a possible association between SARS-CoV-2 exposure and the increased number of cases, potentially accelerating the clinical presentation of type 1 diabetes [3].

From a clinical perspective, a study from Wuhan, China, from October 2020, patients with new-onset diabetes had a higher admission rate to the intensive care unit. These patients required invasive mechanical ventilation and experienced acute kidney injury [4]. Additionally, they had longer hospital stays. The study also reported that elevated blood glucose levels at hospital admission in individuals with new-onset diabetes were associated with an increased risk of all-cause mortality [4].

It remains unclear whether newly diagnosed diabetes associated with COVID-19 is type 1, type 2, or another subtype. The angiotensin-converting enzyme 2 (ACE2) receptor, known as the binding site for SARS-CoV-2, is highly expressed in pancreatic endocrine cells [5]. These observations have raised hypotheses regarding altered glucose metabolism, although limited research exists on islet cell antibodies in individuals with new-onset diabetes, making it difficult to determine the exact mechanism by which SARS-CoV-2 may trigger diabetes [6].

A retrospective single-center medical-recorded review from Alabama evaluated the new-onset diabetes cases from March 2017 to March 2021. During this period, a total of 642 new cases of diabetes were identified. The study observed a significant increase in new-onset type 2 diabetes among Alabama’s young population during the COVID-19 pandemic, highlighting associations rather than causal inference [7].

The last report from the National Center for Nontransmissible Diseases Control and Prevention (CNSCBT) in Romania on SARS-CoV-2 infections stated that 1.813.823 COVID-19 cases and 58.971 deaths (fatality rate: 3.3%) were recorded in 2020 and 2021 [8]. In Romania, in 2021, 800.000 diabetes cases were estimated, mostly type 2, with some reports describing an overlap between SARS-CoV-2 infection and newly detected glycemic abnormalities [9]. Some studies showed that COVID-19 can trigger acute hyperglycemic emergencies, such as diabetic ketoacidosis (DKA) and hyperosmolar hyperglycemic state (HHS), in individuals with newly diagnosed or previously unrecognized diabetes [10].

Therefore, this study aims to describe newly detected dysglycemia and its associated clinical and therapeutic factors in hospitalized COVID-19 patients without prior diabetes.

## 2. Materials and Methods

### 2.1. Study Design and Setting

We collected retrospective COVID-19, register-based data from the Pneumophtisiology Hospital “Leon Daniello” in Cluj-Napoca, Cluj County, Romania. From March 2020 to March 2022, a total of 562 patients, hospitalized during the pandemic COVID-19 peak months, were included in the database. The study population included individuals hospitalized for COVID-19 who did not have a documented diagnosis of type 2 diabetes mellitus prior to admission. Given that HbA1c levels were not available prior to or during hospitalization, the presence of pre-existing diabetes could not be definitively excluded.

**Figure 1:**
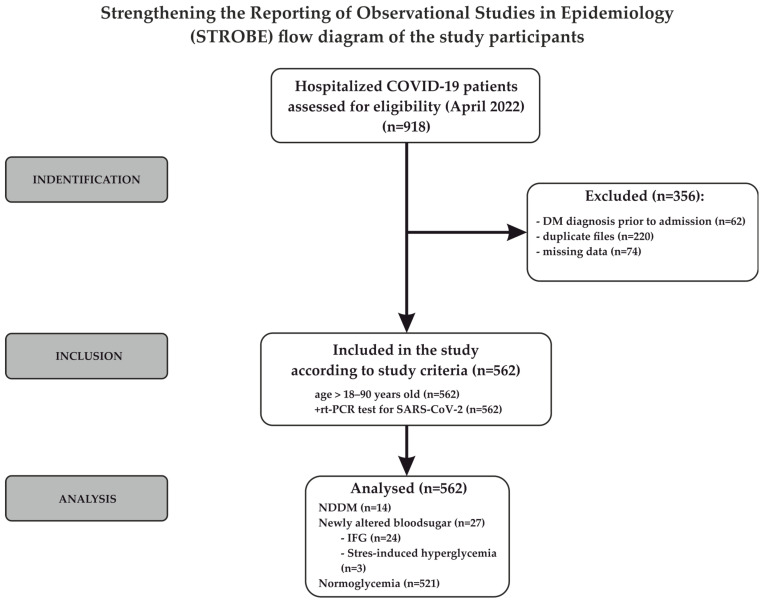
STROBE flow diagram of patient selection in a retrospective study of hospitalized COVID-19 patients evaluating newly detected glycemic alterations, including hyperglycemia and newly diagnosed diabetes; arrows indicate the flow of participants through the study stages, while grey boxes represent the main phases of the study (identification, inclusion, and analysis).

No formal sample size calculation was performed, as the study included all eligible patients identified from the hospital registry. A total of 918 patients were initially identified. After applying predefined exclusion criteria (previous diagnosis of diabetes mellitus, duplicate records, and incomplete clinical or laboratory data), 562 patients were included in the final analysis. The selection of study participants is illustrated in the STROBE flow diagram, created using CorelDRAW (Version 24.0.0.301, 2021) (Figure 1). The reporting method used in this study adhered to the Strengthening the Reporting of Observational Studies in Epidemiology (STROBE) checklist [11]. The completed STROBE checklist is provided as a Supplementary Material.

### 2.2. Participants

The study group included both male and female participants, with a slightly higher proportion of men. Most of the participants were from urban areas. Comorbidities were frequently observed, including cardiovascular conditions, pulmonary diseases, dyslipidemia, hepatic disorders, gastrointestinal diseases, neurological conditions, endocrine disorders, renal disease, and neoplasia. COVID-19 treatment included antiviral agents, such as remdesivir and favipiravir, hydroxychloroquine, and azithromycin; systemic corticosteroids (dexamethasone); and immunomodulatory therapy with tocilizumab. Anticoagulant treatment was also part of the management approach.

The subject’s selection was based on specific eligibility criteria. The inclusion criteria were: age between 18 and 90 years and having a positive rt-PCR test for SARS-CoV-2. No minors were included in the study. The exclusion criteria were: patients who had a diagnosis of diabetes mellitus prior to hospital admission. Also, duplicate files or patients with many missing data were excluded from the research.

The patients were divided into two groups: those with newly diagnosed diabetes mellitus (NDDM) and those with newly altered blood glucose levels. Group allocation was based exclusively on in-hospital plasma glucose measurements. Accordingly, diagnoses of newly diagnosed diabetes or newly altered blood glucose were established during hospitalization based on these measurements.

### 2.3. Data Collection and Variables

Data were collected from electronic health records and patient files. All data were anonymized prior to analysis to ensure patient confidentiality. Patients were identified by the serial number of their medical records. The data were recorded in a secure digital database using Microsoft Excel, with access limited exclusively to the research team. Routine quality control procedures were implemented to ensure data accuracy and completeness.

For each subject, both qualitative and quantitative variables were recorded. These included individual characteristics such sociodemographic data and comorbidities. Clinical data comprised symptoms and vital signs at admission and during hospitalization. Information regarding investigations and COVID-19 treatment was also collected. Associations involving in-hospital treatments were analyzed descriptively and interpreted as observational, without causal inference, given their potential dependence on disease severity and timing during hospitalization. Laboratory data were recorded at admission and during hospitalization. Discharge data included the diagnosis (newly diagnosed diabetes, newly altered blood sugar) and discharge status (cured, improved, stationary, aggravated, deceased). Laboratory parameters were categorized as low, normal, or high based on institutional reference ranges used in routine clinical practice at the study hospital. These reference intervals were applied uniformly for all patients and were consistent with commonly accepted clinical laboratory standards. The exact reference values used to define the normal range for each laboratory parameter are provided in the corresponding table footnotes. Due to the retrospective nature of the study, detailed information on the manufacturers of reagents, kits, and instruments was not available from the recorded data.

### 2.4. Glycemic Classification and Diagnostic Criteria

Glycemic status was assessed using plasma glucose measurements obtained during hospitalization. Blood glucose levels were measured at admission and repeatedly during hospital stay as part of routine clinical care. Newly diagnosed diabetes mellitus was defined according to internationally accepted American Diabetes Association (ADA) criteria [12] (Table 1).

Within the newly altered blood sugar group, among the 27 identified patients, 3 were discharged with a diagnosis of hyperglycemia, while 24 were discharged with a diagnosis of impaired fasting glucose (IFG). IFG was defined according to ADA criteria as fasting plasma glucose levels in the range of 100–125 mg/dL (5.6–6.9 mmol/L). Hyperglycemia was used as a descriptive discharge diagnosis reflecting elevated plasma glucose values during acute illness, likely related to stress-induced hyperglycemia and the use of corticosteroid therapy during COVID-19 hospitalization.

Glycemic classification was confirmed at discharge based on the overall glycemic profile during hospitalization. HbA1c measurements were not available in this retrospective dataset; therefore, classification relied exclusively on in-hospital plasma glucose values.

Patients discharged with newly altered blood sugar levels were referred for post-hospitalization follow-up by diabetologists, where ongoing clinical evaluation and repeat, and glycemic testing were planned to assess the persistence or resolution of glycemic abnormalities. This approach acknowledges that dysglycemia identified during acute COVID-19 illness may reflect stress-related or treatment-associated hyperglycemia rather than established diabetes.

### 2.5. Statistical Analysis

Statistical analysis was performed using the Med Calc^®^ Statistical Software version 23.1.6 (Med Calc Software Ltd., Ostend, Belgium; https://www.medcalc.org; accessed on 10 January 2026). Descriptive and inferential analyses were performed to address the study’s research questions regarding the association among hyperglycemia, newly diagnosed diabetes, and SARS-CoV-2 infection. Continuous variables were summarized using median and interquartile range (non-normal distribution in the Shapiro–Wilk test). Categorical were described using frequencies and percentages. Comparisons between groups regarding qualitative variables were performed using chi-squared test when its assumptions were met, or Fisher’s exact test when appropriate. Comparisons between groups regarding quantitative variables were performed using Mann–Whitney test. A *p*-value < 0.05 was considered statistically significant for all the tests described. For categorical variables, odds ratios (ORs) with 95% confidence intervals (95%CIs) were calculated as unadjusted effect size estimates.

Given the retrospective design and limited number of events, no adjustment for disease severity was performed. Analyses were performed separately for mutually exclusive glycemic subgroups (newly diagnosed diabetes mellitus and newly altered blood sugar). As a result, the number of patients included in specific analyses varies according to the predefined subgroup considered, while the overall cohort size remains unchanged; this variation does not reflect missing data.

### 2.6. Ethical Consideration

The study protocol was approved by the Ethics Review Board (AVZ90, Approval Date: 13 April 2022) of UMF Iuliu Hatieganu Cluj-Napoca, and was conducted according to the guidelines of the Declaration of Helsinki. All patients or their legal representatives were informed upon admission that medical data may be used for research purposes, in accordance with institutional and national regulations. Given the retrospective, register-based data of the study, informed consent was waived where applicable. Personal data confidentiality was respected by anonymizing it within the study.

## 3. Results

### 3.1. Normal vs. Newly Diagnosed Diabetes

#### 3.1.1. COVID-19 Vaccination Status Stratified by Period

Among newly diagnosed diabetes patients admitted between October and December 2020, four of them were documented as unvaccinated, coresponding to the pre-vaccination period. During the March–May 2021 period, vaccination status was unavailable for nine patients. In February 2022, one patient was documented as unvaccinated.

#### 3.1.2. General Characteristics and Comorbidities

Out of the total number of 562 patients, 14 (2.49%) were newly diagnosed with diabetes. Among these, 57.14% were male and 42.86% were female. The median age of the participants was 67.5 years (IQR: 59.75;71.75). In terms of the environment of origin, 64.29% come from urban areas and 35.71% come from rural areas. All of them had known comorbidities, with cardiovascular conditions being the most common, affecting 64.29% of the patients (Table 2). As the number of patients with newly diagnosed diabetes is very small, all ORs were associated with wide/very wide 95%CIs, and none of the investigated associations achieved statistical significance. These analyses should be interpreted just as exploratory and descriptive.

#### 3.1.3. Symptoms at Admission

Regarding admission to the Pneumophthisiology Department, patients with newly diagnosed diabetes presented dyspnea (*p* = 0.085) or other symptoms (*p* = 0.077). These values did not reach statistical significance (*p* > 0.05). Given the small size of the newly diagnosed diabetes subgroup (n = 14), the observed association between diarrhea and newly diagnosed diabetes, although statistically significant (*p* < 0.001), should be interpreted with caution, as the odds ratio may be unstable (Table 3).

#### 3.1.4. Investigations and Treatment

Patients with newly diagnosed diabetes were more likely to receive oxygen therapy, but the difference did not reach statistical significance (*p* = 0.091). For corticosteroid therapy, particularly with dexamethasone (*p* = 0.007), we found a *p*-value that did not reach statistical significance, but with a very wide 95%CI, reflecting the low number of patients with diabetes. An observational association (*p* = 0.02) was identified between remdesivir use and newly diagnosed diabetes. This finding reflects in-hospital treatment exposure and should be interpreted descriptively. Regarding anticoagulant therapy, patients with newly diagnosed diabetes were more likely to receive this therapy, but no statistically significant association was observed (*p* = 0.084) (Table 4).

#### 3.1.5. Laboratory Results at Admission and During Hospitalization

For the medical tests, at admission, we found a statistically significant *p*-value for procalcitonin (*p* = 0.015). For C-reactive protein (CRP), no statistically significant association was observed (*p* = 0.081) (Table 5).

#### 3.1.6. Age, Days of Hospitalization, Heart Rate, and Oxygen Saturation

Regarding differences in age, hospitalization days, heart rate, or oxygen saturation between patients without diabetes and those newly diagnosed with diabetes, no statistically significant correlation was found. However, newly diagnosed diabetes patients tended to be older, had slightly longer hospital stays, and had higher heart rates with lower oxygen saturation levels (Table 6).

### 3.2. Normal Blood Sugar vs. Newly Altered Blood Sugar

#### 3.2.1. COVID-19 Vaccination Status Stratified by Period

Among patients with newly altered blood sugar levels, one patient admitted between March and May 2020 and nine patients admitted between October and December 2020 were documented as unvaccinated corresponding to the pre-vaccination period. During the March–April 2021 period, vaccination status was unavailable for 10 patients. In January 2022, one patient was documented as vaccinated, while in February 2022, one patient was unvaccinated and vaccination status was unavailable for three patients. In March 2022, two patients were documented as unvaccinated.

#### 3.2.2. General Characteristics and Comorbidities

From the total number of patients, 27 (4.8%) were diagnosed with newly altered blood sugar. Among these, 52.96% were men, and 37.04% were women. The median age of the participants was 65 (IQR: 49.5;71). Regarding their background, 59.26% come from urban areas and 40.76% from rural areas. Only one patient had been COVID-19 vaccinated. A total of 24 patients had known comorbidities, with cardiovascular conditions being the most common, affecting 59.26% of the patients.

Among comorbidities, dyslipidemia (*p* = 0.021) and neurological pathologies (*p* = 0.046) were statistically significant associated with newly altered blood sugar (Table 7).

#### 3.2.3. Symptoms at Admission

Regarding the clinical picture at admission to the Pneumophtisiology Department, patients with newly altered blood sugar were more likely to present sweating; however, this difference did not reach statistical significance (*p* = 0.059). A significant difference was found for dyspnea (*p* = 0.002). Dispneea was more common among patients with newly altered blood sugar (17 patients) compared with those with normal blood sugar (174 patients). However, the clinical relevance of this difference should be interpreted cautiously (Table 8).

#### 3.2.4. Investigations and Treatment

Investigations were performed on all patients, including 25 chest scans, 1 chest X-ray, and 27 ECGs. Regarding COVID-19 treatment, 3 patients received remdesivir, 10 received favipiravir, 6 received hydroxychloroquine, 1 received azithromycin, 24 received corticosteroid/dexamethasone, 26 received anticoagulant, and 1 received insulin. Patients with newly altered blood sugar were more likely to receive oxygen therapy, with a statistically significant level (*p* = 0.015). An observational association between COVID-19 treatments with favipiravir (*p* = 0.025), corticosteroid therapy, particularly with dexamethasone (*p* = 0.02) and anticoagulants (*p* = 0.042) and elevated glucose levels was observed (Table 9).

#### 3.2.5. Laboratory Tests at Admission and During Hospitalization

For the medical tests, we found a statistically significant *p*-value for d-dimer (D-dimer-1) at admission (*p* = 0.004) (Table 10).

#### 3.2.6. Age, Days of Hospitalization, Heart Rate, and Oxygen Saturation

There is a statistically significant difference between the groups in terms of hospitalization days (*p* = 0.035). However, the absolute difference in length of stay was relatively small and should be interpreted with caution to avoid overestimating its clinical relevance. Age, heart rate, and oxygen saturation did not show significant differences between patients with normal blood sugar levels and those with newly altered blood sugar levels (Table 11).

## 4. Discussion

This study explores the association between COVID-19, newly altered blood sugar, and newly diagnosed diabetes. Additionally, we highlight various sociodemographic and medical factors that may precipitate glycemic changes. In our cohort, remdesivir and favipiravir use was observationally associated with elevated glucose levels. However, this association may reflect confounding by indication, as remdesivir was more frequently administered to patients with more severe disease, who are themselves at increased risk of stress-related hyperglycemia. Therefore, these findings should be interpreted as associative rather than causal.

The medical literature suggests that viral infections or other acute illnesses can be temporally associated with the onset of diabetes mellitus. Studies indicate that SARS-CoV-2 infection may be linked to alteration in pancreatic function, insulin resistance, and glucose metabolism, thereby being associated with the risk of developing diabetes or experiencing glycemic changes [13].

About newly altered blood glucose levels group, statistically significant associations were found with comorbidities such as dyslipidemia and neurological diseases. In a study that evaluated the relationship between newly diagnosed diabetes, hyperglycemia, the atherogenic index, and obesity in post-COVID-19 syndrome, researchers identified a potential interplay between metabolic dysfunction and the emergence of post-COVID-19 glycemic disturbances [14].

In the newly diagnosed diabetes group, diarrhea had the most statistically significant difference. Additionally, dyspnea at admission was significantly associated with newly altered blood sugar. Patients from both groups had a higher need for oxygen therapy. In a retrospective study, analyzing symptoms at the time of COVID-19 first hospitalization, dry cough, fever, and dyspnea were the most commonly observed symptoms [15]. Another cross-sectional study supports our findings, highlighting diarrhea and dyspnea as possible onset symptoms in newly diagnosed diabetes, emphasizing the importance of monitoring both respiratory and gastrointestinal symptoms [16]. These findings align with previous research indicating that newly diagnosed diabetes and hyperglycemia at hospital admission are associated with rapid respiratory decline in patients with COVID-19 [17].

Patients with newly diagnosed diabetes who experienced a more severe form of COVID-19 required treatment with corticosteroids and remdesivir, presented with elevated procalcitonin levels upon admission, and showed increased levels of both C-reactive protein and procalcitonin on follow-up evaluations. Several studies in the literature have described an observational association between remdesivir administration and increased glucose levels [18,19,20]. However, these findings are not in agreement with a Japanese case report, which describes remdesivir-induced hypoglycemia in an elderly man without a prior diagnosis of diabetes [21].

Our findings suggest that, across both groups, the administration of corticosteroids may be associated with increased blood sugar levels. Favipiravir and anticoagulants may influence glycemic fluctuations observed during hospitalization in newly diagnosed diabetes group. It is known that glucocorticoid therapy can be accompanied by hyperglycemia or steroid-induced diabetes mellitus [22,23,24]. Regarding favipiravir treatment, these finding contrasts with the results of a retrospective study that aimed to determine the adverse effects of favipiravir administration in COVID-19 patients. In that study, the side effects of favipiravir included elevated transaminases, gastrointestinal symptoms, high blood sugar, and thrombocytopenia. The distribution of adverse effects for the high blood sugar level was only 5% [25]. Meanwhile, other studies highlight the association between favipiravir and theophylline, which may be related to increased blood sugar levels [26].

Furthermore, we found an association between elevated D-dimer values upon admission and newly altered blood glucose levels. Laboratory tests showed elevated levels of some inflammatory markers, such as procalcitonin, at admission and during hospitalization, and CRP during hospitalization. These findings emphasize the importance of considering the severity of the SARS-CoV-2 infection and its association with the risk of glycemic disturbances, including the onset of newly diagnosed diabetes. Inflammatory markers such as CRP appear to be more commonly encountered in COVID-19 infection in NDDM (newly diagnosed diabetes mellitus) [5].

Moreover, we found a statistically significant difference between the groups in terms of hospitalization days. Data from our study showed that most of the patients from both, newly diagnosed group and newly altered blood sugar, are older, with a statistically significant difference between the groups in terms of hospitalization days for the newly altered blood sugar group. A review on the medical factors influencing COVID-19 severity noted that diabetes is associated with worse outcomes in COVID-19 patients, resulting in increased hospitalization and higher rates of ICU admissions. This highlights the vulnerability of diabetic patients in the face of COVID-19 [17,27]. Also, several studies have shown impaired pulmonary function correlates with blood glucose levels [28].

Among the multitude of studies that have evaluated the relationship between COVID-19 and blood glucose changes, including diabetes mellitus, some have emphasized the importance of distinguishing between newly diagnosed and new-onset diabetes. A review article from 2023 stated that newly diagnosed diabetes refers to diabetes that was previously present but undetected, whereas new-onset diabetes refers to the absence of any prior diabetes [29].

SARS-CoV-2 infection spread rapidly, becoming a major public health problem. Authorities and scientists have worked to find the best preventive measures to protect people from contracting the disease. Vaccines have been developed, safety measures implemented, and access to education has been prioritized, as it is an essential component of public health. From a clinical perspective, the observed associations underscore the need for careful glycemic assessment in hospitalized patients with COVID-19. Integrating routine glucose monitoring into inpatient management may facilitate early recognition of dysglycemia and inform appropriate post-discharge metabolic follow-up. The observed glycemic alterations during hospitalization may also reflect early manifestations of post-acute COVID-19 metabolic sequelae. Emerging evidence suggests that long COVID may contribute to sustained dysregulation of glucose metabolism, potentially precipitating new-onset diabetes or worsening pre-existing metabolic vulnerability [30,31]. These findings highlight the importance of longitudinal metabolic monitoring in individuals recovering from COVID-19.

Our study has some limitations. The study was conducted in a single hospital and has a retrospective design. The reliance on existing medical records may lead to incomplete or missing data. The absence of pre-admission HbA1c measurements limited the ability to differentiate between previously unrecognized diabetes and dysglycemia detected during COVID-19 hospitalization; therefore, the diagnosis of newly diagnosed diabetes and newly altered blood sugar was confirmed only at discharge. Additionally, we acknowledge that COVID-19 severity and treatment intensity may have acted as important confounding factors in the observed associations. Due to the limited number of patients diagnosed with NDDM (n = 14), multivariable logistic regression analysis was not performed for this outcome in order to avoid model overfitting and unstable estimates. Therefore, analyses related to NDDM were restricted to descriptive statistics and exploratory comparisons. The absence of a non-COVID-19 control group precludes causal inference regarding the role of SARS-CoV-2 infection in the development of diabetes. Therefore, the findings should be interpreted as descriptive and associative rather than causal. Vaccination status was not included in comparative statistical analyses, due to substantial missing data and calendar-time heterogeneity. These limitations emphasize the need for future observational studies to validate and confirm these findings.

The study’s strength is that it provides additional information regarding the possible involvement of COVID-19, sociodemographic, and medical factors in the occurrence of glycemic changes.

## 5. Conclusions

Among hospitalized COVD-19 patients, corticosteroid therapy, and the presence of one or more chronic conditions, such as cardiovascular disease or dyslipidemia, was observationally associated with glycemic alterations during hospitalization. Careful monitoring of admission symptoms, laboratory parameters, oxygen requirements, and sociodemographic characteristics may help identify patients at higher risk of developing dysglycemia during acute COVID-19 illness. Also, glycemic alterations were observed among patients treated with remdesivir and favipiravir; however, this association should be interpreted cautiously, as it may reflect confounding by disease severity rather than a direct effect on glucose metabolism.

Despite temporal variations in incidence, COVID-19 remains an important public health concern, with implications for quality of life, economics, and human resources. Data from future studies may help create and implement new strategies to evaluate and keep under observation patients addressed to the hospital for COVID-19, particularly those with one or more risk factors for developing diabetes.

## Figures and Tables

**Table 1:** Diagnostic criteria for diabetes in non-pregnant adults.

Diagnostic Test		Criteria
Glycated hemoglobin		≥6.5% (≥48 mmol/mol); performed using an NGSP-certified method standardized to the DCCT assay *
	or	
Fasting plasma glucose (FPG)		≥126 mg/dL (≥7.0 mmol/L); fasting defined as no caloric intake for at least 8 h *
	or	
2-hour plasma glucose (2-hour PG) during OGTT		≥200 mg/dL (11.1 mmol/L) during an OGTT. The test should be performed as described by the World Health Organization, using a glucose load containing the equivalent of 75 g anhydrous glucose dissolved in water *
	or	
Random plasma glucose		≥200 mg/dL (≥11.1 mmol/L) in individuals with classic symptoms of hyperglycemia or hyperglycemic crisis

DCCT: Diabetes Control and Complications Trial; FPG: fasting plasma glucose; NGSP: National Glycohemoglobin Standardization Program; OGTT: oral glucose tolerance test. * In the absence of unequivocal hyperglycemia, diagnosis requires 2 abnormal tests results from the same sample or in 2 separate test samples. Reproduced with permission from: American Diabetes Association. Standards of Medical Care in Diabetes 2025 [12].

**Table 2:** Normal vs. newly diagnosed diabetes. General characteristics, COVID-19 status, and medical history.

Variable		Total—n (%)	Without Diabetes—n (%)	Newly Diagnosed Diabetes—n (%)	OR (95% CI)	*p*
gender	female	258 (48.22)	252 (48.37)	6 (42.86)	1.25 (0.43–3.64)	0.790 *
	male	277 (51.78)	269 (51.63)	8 (57.14)		
origin	rural	155 (29.03)	150 (28.85)	5 (35.71)	1.00 (0.33–3.04)	1 *
	urban	379 (70.97)	270 (71.15)	9 (64.29)		
comorbidities		448 (83.74)	434 (83.3)	14 (100)	6.06 (0.36–101.3)	0.141 **
renal		57 (10.65)	56 (10.75)	1 (7.14)	0.64 (0.08–5.19)	1 *
hepatic		86 (16.1)	82 (15.77)	4 (28.57)	2.13 (0.65–6.97)	0.257 **
cardiovascular		286 (53.46)	277 (53.17)	9 (64.29)	1.58 (0.53–4.67)	0.41 **
dyslipidemia		102 (19.14)	98 (18.88)	4 (28.57)	1.71 (0.52–5.58)	0.32 **
pulmonary		189 (35.46)	183 (35.26)	6 (42.86)	1.38 (0.47–4.08)	0.579 **
endocrine		60 (11.24)	60 (11.54)	0 (0)	0.26 (0.02–4.60)	0.385 *
gastrointestinal		84 (15.73)	82 (15.77)	2 (14.29)	0.89 (0.20–4.03)	1 *
neoplasia		45 (8.72)	44 (8.75)	1 (7.69)	0.84 (0.11–6.68)	1 *
neurological		109 (20.45)	108 (20.85)	1 (7.14)	0.31 (0.04–2.46)	0.32 *
COVID-19 contact		41 (60.29)	40 (59.7)	1 (100)	6.06 (0.36–101.3)	1 *

Percentages are calculated within each subgroup. * Fisher’s exact test; ** Chi-squared test; *p* < 0.05 was considered statistically significant.

**Table 3:** COVID-19 patients. Normal vs. newly diagnosed diabetes. Symptoms at admission.

Variable	Total—n (%)	Without Diabetes—n (%)	Newly Diagnosed Diabetes—n (%)	OR (95%CI)	*p*
cough	288 (53.93)	278 (53.46)	10 (71.43)	2.13 (0.66–6.86)	0.183 *
fever	94 (17.67)	92 (17.76)	2 (14.29)	0.77 (0.17–3.43)	1 *
headache	108 (20.22)	108 (20.77)	0 (0)	0.16 (0.01–2.86)	0.084 *
fatigue	94 (17.6)	93 (17.88)	1 (7.14)	0.35 (0.04–2.66)	0.482 *
dyspnea	182 (34.08)	174 (33.46)	8 (57.14)	2.63 (0.89–7.79)	0.085 **
sweating	28 (5.24)	27 (5.19)	1 (7.14)	1.42 (0.18–11.3)	0.534 *
diarrhea	29 (5.43)	24 (4.62)	5 (35.71)	11.46 (3.36–39.1)	<0.001 *
ageusia	33 (6.18)	33 (6.35)	0 (0)	0.49 (0.03–8.87)	1 *
anosmia	38 (7.13)	38 (7.32)	0 (0)	0.42 (0.02–7.64)	0.613 *
other symptoms	378 (70.92)	365 (70.33)	13 (92.86)	5.43 (0.71–41.5)	0.077 *

Percentages are calculated within each subgroup. * Fisher’s exact test; ** Chi-squared test; *p* < 0.05 was considered statistically significant.

**Table 4:** COVID-19 patients. Normal vs. newly diagnosed diabetes. Investigations and treatment.

Variable	Total—n (%)	Without Diabetes—n (%)	Newly Diagnosed Diabetes—n (%)	OR (95%CI)	*p*
investigations	528 (99.62)	514 (99.61)	14 (100)	0.42 (0.02–7.76)	1 *
chest tomography	436 (81.8)	427 (82.27)	9 (64.29)	0.41 (0.12–1.41)	0.149 *
chest X-ray	92 (17.23)	89 (17.12)	3 (21.43)	1.33 (0.36–4.98)	0.718 *
ECG	534 (99.81)	520 (99.81)	14 (100)	1.33 (0.36–4.98)	1 *
ICU pneumology department	35 (6.54)	33 (6.33)	2 (14.29)	2.47 (0.53–11.6)	0.231 *
administration of oxygen	262 (49.16)	252 (48.55)	10 (71.43)	2.66 (0.82–8.60)	0.091 *
COVID-19 treatment	484 (90.64)	470 (90.38)	14 (100)	3.37 (0.20–56.9)	0.383 *
remdesivir	43 (8.1)	39 (7.54)	4 (28.57)	4.94 (1.48–16.5)	0.021 *
favipiravir	102 (19.17)	100 (19.31)	2 (14.29)	0.72 (0.16–3.31)	1 *
hydroxychloroquine	170 (32.02)	166 (32.11)	4 (28.57)	0.85 (0.27–2.70)	1 *
azithromycin	49 (9.21)	49 (9.46)	0 (0)	0.36 (0.02–6.49)	0.629 *
cortico-dexamethasone	364 (68.42)	350 (67.57)	14 (100)	8.48 (0.50–143.5)	0.007 *
tocilizumab	4 (0.75)	4 (0.77)	0 (0)	3.54 (0.18–71.1)	1 *
anticoagulant	435 (81.61)	421 (81.12)	14 (100)	5.90 (0.35–99.6)	0.084 *

ECG = Electrocardiogram. Percentages are calculated within each subgroup. * Fisher’s exact test; *p* < 0.05 was considered statistically significant.

**Table 5:** COVID-19 patients. Normal vs. newly diagnosed diabetes. Medical test: 1 admission, 2 during hospitalization.

Variable	Item	Total—n (%)	Without Diabetes—n (%)	Newly Diagnosed Diabetes—n (%)	OR (95%CI)	*p*
ESR-1	Normal	115 (33.05)	114 (33.43)	1 (14.29)	3.01 (0.36–25.3)	0.433 *
	High	233 (66.95)	227 (66.57)	6 (85.71)		
WBC-1	Low	92 (18.55)	91 (18.84)	1 (7.69)	2.86 (0.86–9.53)	0.177 *
	Normal	335 (67.54)	327 (67.7)	8 (61.54)		
	High	69 (13.91)	65 (13.46)	4 (30.77)		
Ferritin-1	Low	3 (0.71)	3 (0.73)	0 (0)	1.17 (0.24–5.64)	1 *
	Normal	115 (27.32)	113 (27.36)	2 (25)		
	High	303 (71.97)	297 (71.91)	6 (75)		
CRP-1	Normal	105 (21.88)	105 (22.48)	0 (0)	7.72 (0.45–132.7)	0.081 *
	High	375 (78.12)	362 (77.52)	13 (100)		
D-dimer-1	Normal	252 (66.32)	245 (66.4)	7 (63.64)	1.13 (0.33–3.93)	1 *
	High	128 (33.68)	124 (33.6)	4 (36.36)		
PCT-1	Normal	241 (62.76)	239 (63.73)	2 (22.22)	6.14 (1.28–29.5)	0.015 *
	High	143 (37.24)	136 (36.27)	7 (77.78)		
WBC-2	Low	15 (4.39)	15 (4.55)	0 (0)	0.90 (0.27–3.02)	1 *
	Normal	205 (59.94)	197 (59.7)	8 (66.67)		
	High	122 (35.67)	118 (35.76)	4 (33.33)		
CRP-2	Normal	147 (46.67)	142 (46.86)	5 (41.67)	1.23 (0.40–3.82)	0.723 **
	High	168 (53.33)	161 (53.14)	7 (58.33)		
D-dimer-2	Normal	150 (58.14)	144 (58.3)	6 (54.55)	1.16 (0.35–3.87)	1 *
	High	108 (41.86)	103 (41.7)	5 (45.45)		
LDH-2	Normal	225 (73.05)	217 (73.31)	8 (66.67)	1.37 (0.41–4.57)	0.74 **
	High	83 (26.95)	79 (26.69)	4 (33.33)		
PCT-2	Normal	126 (52.07)	124 (53.22)	2 (22.22)	3.99 (0.83–19.3)	0.091 *
	High	116 (47.93)	109 (46.78)	7 (77.78)		
Ferritin-2	Normal	61 (20.27)	58 (20)	3 (27.27)	0.67 (0.18–2.55)	0.723 **
	High	238 (79.07)	230 (79.31)	8 (72.73)		
	Low	2 (0.66)	2 (0.69)	0 (0)		
ESR-2	Normal	46 (33.09)	46 (33.82)	0 (0)	3.59 (0.18–70.8)	0.551 *
	High	93 (66.91)	90 (66.18)	3 (100)		

1 = admission; 2 = during hospitalization; ESR = Erythrocyte Sedimentation Rate (males: 3–8 mm/h; females: 6–11 mm/h); WBC = White Blood Cell (4.2–10 × 10^3^/μL); CRP = C-reactive protein (0–5 mg/L); PCT = Procalcitonin (<0.5 ng/mL—low risk of sepsis; >0.5 ng/mL—increased risk of sepsis); LDH = Lactate Dehydrogenases (125–220 U/L); Ferritin (males: 30–400 ng/mL; females: 15–150 ng/mL); D-dimer (0–500 ng/mL). Percentages are calculated within each subgroup. * Fisher’s exact test; ** Chi-squared test; *p* < 0.05 was considered statistically significant.

**Table 6:** COVID-19 patients. Normal vs. newly diagnosed diabetes. Comparison of clinical parameters.

Variable	Total—Median (IQR)	Without Diabetes—n (%)	Newly Diagnosed Diabetes—n (%)	*p*
age	57 (44;70)	57 (44;70)	67.5 (59.75;71.75)	0.091
hospitalization days	11 (7;14.25)	11 (7;14.25)	12.5 (9.25;14.75)	0.641
heart rate	83 (74;94)	83 (74;94)	90 (83;99)	0.379
oxygen saturation	96 (93;98)	96 (93;98)	94 (91;96)	0.461

IQR = Interquartile Range. Percentages are calculated within each subgroup. chi-squared test; *p* < 0.05 was considered statistically significant.

**Table 7:** COVID-19 patients. Normal blood sugar vs. newly altered blood sugar. General characteristics, COVID-19 status, and medical history.

Variable		Total—n (%)	Normal Blood Sugar—n (%)	Newly Altered Blood Sugar—n (%)	OR (95%CI)	*p*
gender	female	262 (47.81)	252 (48.37)	10 (37.04)	1.59 (0.72–3.54)	0.25 **
	male	286 (52.19)	269 (51.63)	17 (62.96)		
origin	rural	161 (29.43)	150 (28.859	11 (40.74)	1.70 (0.77–3.75)	0.186 **
	urban	386 (70.579	370 (71.15)	16 (59.26)		
comorbidities		458 (83.58)	434 (83.3)	24 (88.89)	1.60 (0.47–5.44)	0.598 *
renal		59 (10.77)	56 (10.75)	3 (11.11)	1.04 (0.30–3.56)	1 *
hepatic		86 (15.72)	82 (15.77)	4 (14.81)	0.93 (0.31–2.76)	1 *
cardiovascular		293 (53.47)	277 (53.17)	16 (59.26)	1.28 (0.58–2.81)	0.536 **
dyslipidemia		108 (19.78)	98 (18.88)	10 (37.04)	2.54 (1.13–5.72)	0.021 *
pulmonary		193 (35.35)	183 (35.26)	10 (37.04)	1.09 (0.49–2.42)	0.851 **
endocrine		63 (11.52)	60 (11.54)	3 (11.11)	0.96 (0.28–3.29)	1 *
gastrointestinal		88 (16.09)	82 (15.77)	6 (22.22)	1.53 (0.60–3.91)	0.417 *
neoplastic		46 (8.71)	44 (8.75)	2 (8)	0.87 (0.20–3.78)	1 *
neurological		118 (21.61)	108 (20.85)	10 (37.04)	2.25 (1.00–5.05)	0.04 **

Percentages are calculated within each subgroup. * Fisher test, ** Chi-squared test; *p* < 0.05 was considered statistically significant.

**Table 8:** COVID-19 patients. Normal blood sugar vs. newly altered blood sugar. Symptoms at admission.

Variable	Total—n (%)	Normal Blood Sugar—n (%)	Newly Altered Blood Sugar—n (%)	OR (95%CI)	*p*
cough	297 (54.3)	278 (53.46)	19 (70.37)	2.07 (0.91–4.71)	0.85 **
fever	96 (17.61)	92 (17.76)	4 (14.81)	0.81 (0.28–2.36)	1 *
headache	112 (20.48)	108 (20.77)	4 (14.81)	0.66 (0.23–1.92)	0.455 *
fatigue	101 (18.46)	93 (17.88)	8 (29.63)	1.93 (0.84–4.43)	0.13 **
dyspnea	191 (34.92)	174 (33.46)	17 (62.96)	3.39 (1.52–7.56)	0.002 **
sweating	31 (5.67)	27 (5.19)	4 (14.81)	3.19 (0.99–10.3)	0.059 *
diarrhea	24 (4.39)	24 (4.62)	0 (0)	0.36 (0.02–6.33)	0.623 *
ageusia	36 (6.58)	33 (6.35)	3 (11.11)	1.84 (0.52–6.46)	0.411 *
anosmia	40 (7.33)	38 (7.32)	2 (7.41)	1.02 (0.23–4.48)	1 *
other symptoms	382 (69.96)	365 (70.33)	17 (62.96)	0.71 (0.32–1.57)	0.416 **

Percentages are calculated within each subgroup. * Fisher test, ** Chi-squared test; *p* < 0.05 was considered statistically significant.

**Table 9:** COVID-19 patients. Normal blood sugar vs. newly altered blood sugar. Investigations and treatment.

Variable	Total—n (%)	Normal Blood Sugar—n (%)	Newly Altered Blood Sugar—n (%)	OR (95%CI)	*p*
investigations	541 (99.63)	514 (99.61)	27 (100)	0.42 (0.02–7.76)	1 *
chest tomography	452 (82.94)	427 (82.27)	25 (96.15)	5.38 (1.23–23.5)	0.103 *
chest X-ray	90 (16.45)	89 (17.12)	1 (3.7)	0.19 (0.02–1.44)	0.104 *
ECG	547 (99.82)	520 (99.81)	27 (100)	1.05 (0.04–26.1)	1 *
ICU pneumology department	34 (6.2)	33 (6.339)	1 (3.7)	0.56 (0.07–4.32)	1 *
administration of oxygen	271(49.72)	252 (48.55)	19 (73.08)	2.90 (1.18–7.12)	0.015 **
COVID-19 treatment	497 (90.86)	470 (90.38)	27 (100)	5.93 (0.35–100.1)	0.16 *
remdesivir	42 (7.72)	39 (7.54)	3 (11.11)	1.54 (0.45–5.29)	0.455 **
favipiravir	110 (20.18)	100 (19.31)	10 (37.04)	2.48 (1.10–5.57)	0.025 **
hydroxychloroquine	172 (31.62)	166 (32.11)	6 (22.22)	0.61 (0.24–1.55)	0.281 **
azithromycin	50 (9.17)	49 (9.46)	1 (3.7)	0.36 (0.05–2.70)	0.498 *
cortico-dexamethasone	374 (68.62)	350 (67.57)	24 (88.89)	3.91 (1.16–13.16)	0.02 **
tocilizumab	4 (0.73)	4 (0.77)	0 (0)	2.11 (0.11–40.3)	1 *
anticoagulant	447 (81.87)	421 (81.12)	26 (96.3)	5.76 (0.77–43.2)	0.042 *
insulin	8 (1.47)	7 (1.35)	1 (3.7)	2.80 (0.34–23.1)	0.336 *

ECG = Electrocardiogram; Percentages are calculated within each subgroup. * Fisher test, ** Chi-squared test; *p* < 0.05 was considered statistically significant.

**Table 10:** COVID-19 patients. Normal blood sugar vs. newly altered blood sugar. Laboratory tests: 1 admission; 2 during hospitalization.

Variable	Item	Total—n (%)	Normal Blood Sugar—n (%)	Newly Altered Blood Sugar—n (%)	OR (95%CI)	*p*
ESR-1	Normal	120 (33.71)	114 (33.43)	6 (40)	0.75 (0.26–2.19)	0.598 **
	High	236 (66.29)	227 (66.57)	9 (60)		
WBC-1	Low	96 (18.82)	91 (18.84)	5 (18.52)	1.46 (0.53–4.01)	0.723 *
	Normal	344 (67.45)	327 (67.7)	17 (62.96)		
	High	70 (13.73)	65 (13.46)	5 (18.52)		
Ferritin-1	Low	3 (0.69)	3 (0.73)	0 (0)	1.48 (0.53–4.15)	0.694 *
	Normal	118 (27)	113 (27.36)	5 (20.83)		
	High	316 (72.31)	297 (71.91)	19 (79.17)		
CRP-1	Normal	108 (21.91)	105 (22.48)	3 (11.54)	2.22 (0.64–7.69)	0.189 *
	High	385 (78.09)	362 (77.52)	23 (88.46)		
	High	26 (66.67)	25 (67.57)	1 (50)		
D-dimer-1	Normal	252 (64.78)	245 (66.4)	7 (35)	3.35 (1.45–7.74)	0.004 **
	High	137 (35.22)	124 (33.6)	13 (65)		
PCT-1	Normal	252 (63.96)	239 (63.73)	13 (68.42)	0.67 (0.04–10.9)	0.678 *
	High	142 (36.04)	136 (36.27)	6 (31.58)		
WBC-2	Low	16 (4.58)	15 (4.55)	1 (5.26)	1.61 (0.62–4.19)	0.428 *
	Normal	206 (59.03)	197 (59.7)	9 (47.37)		
	High	127 (36.39)	118 (35.76)	9 (47.37)		
CRP-2	Normal	152 (46.91)	142 (46.86)	10 (47.62)	0.97 (0.39–2.41)	0.947 **
	High	172 (53.09)	161 (53.14)	11 (52.38)		
D-dimer-2	Normal	155 (58.05)	144 (58.3)	11 (55)	1.14 (0.45–2.91)	0.774 **
	High	112 (41.95)	103 (41.7)	9 (45)		
LDH-2	Normal	234 (73.58)	217 (73.31)	17 (77.27)	0.81 (0.29–2.30)	0.684 **
	High	84 (26.42)	79 (26.69)	5 (22.73)		
PCT-2	Normal	133 (53.85)	124 (53.22)	9 (64.29)	0.63 (0.20–1.99	0.42 *
	High	114 (46.15)	109 (46.78)	5 (35.71)		
	High	25 (8.17)	23 (8.07)	2 (9.52)		
Ferritin-2	Low	2 (0.64)	2 (0.69)	0 (0)	1.74 (0.50–6.03)	0.645 *
	Normal	61 (19.49)	58 (20)	3 (13.04)		
	High	250 (79.87)	230 (79.31)	20 (86.96)		
ESR-2	Normal	49 (34.27)	46 (33.82)	3 (42.86)	0.68 (0.14–3.33)	0.691 *
	High	94 (65.73)	90 (66.18)	4 (57.14)		

1 = admission; 2 = during hospitalization; ESR = Erythrocyte Sedimentation Rate (males: 3–8 mm/h; females: 6–11 mm/h); WBC = White Blood Cell (4.2–10 × 10^3^/μL); CRP = C-reactive protein (0–5 mg/L); PCT = Procalcitonin (<0.5 ng/mL—low risk of sepsis; >0.5 ng/mL—increased risk of sepsis); LDH = Lactate Dehydrogenases (125–220 U/L); Ferritin (males: 30–400 ng/mL, females: 15–150 ng/mL); D-dimer (0–500 ng/mL). Percentages are calculated within each subgroup. * Fisher test, ** Chi-squared test; *p* < 0.05 was considered statistically significant.

**Table 11:** COVID-19 patients. Normal blood sugar vs. newly altered blood sugar. Comparison of clinical parameters.

Variable	Total—Median (IQR)	Normal Blood Sugar—Median (IQR)	Newly Altered Blood Sugar—Median (IQR)	*p*
age	57 (45;70)	57 (44;70)	65 (49.5;71)	0.101
hospitalization days	11 (7;14.5)	11 (7;14;25)	12 (10;14.5)	0.035
heart rate	82 (74;94)	83 (74;94)	82 (76;90)	0.601
oxygen saturation	96 (93;98)	96 (93;97)	96 (92;97)	0.643

IQR = Interquartile Range. Percentages are calculated within each subgroup. chi-squared test; *p* < 0.05 was considered statistically significant.

## Data Availability

The data generated and analyzed in this study are not publicly available, as they were specifically collected for the purposes of this research. They can be made available by the corresponding author upon reasonable request.

## Supplementary Materials

The following supporting information can be downloaded at: https://www.mdpi.com/article/10.3390/epidemiologia7020054/s1, STROBE Statement—Checklist. The completed STROBE checklist confirms adherence to STROBE guidelines and reports key aspects of study design, methods, and analysis, ensuring transparent reporting.

## Abbreviations

IDF	International Diabetes Federation
WHO	World Health Organization
ACE2	angiotensin-converting enzyme 2
CNSCBT	National Center for Nontransmissible Diseases Control and Prevention
DKA	diabetic ketoacidosis
HHS	hyperosmolar hyperglycemic state
NDDM	newly diagnosed diabetes mellitus
